# Proteinuria and hematuria as early signs of renal involvement in juvenile idiopathic arthritis

**DOI:** 10.3389/fped.2024.1395961

**Published:** 2024-06-04

**Authors:** Emanuela Del Giudice, Alessia Marcellino, Sara Hoxha, Vanessa Martucci, Mariateresa Sanseviero, Silvia Bloise, Sara Giovanna De Maria, Maria Rita Leone, Flavia Ventriglia, Riccardo Lubrano

**Affiliations:** Pediatrics and Neonatology Unit, Department of Maternal Infantile and Urological Sciences, Sapienza University of Rome, Polo Pontino, Santa Maria Goretti Hospital, Latina, Italy

**Keywords:** blood pressure, glomerular function, hematuria, juvenile idiopathic arthritis, tubular renal function, pediatric proteinuria

## Abstract

**Objectives:**

To evaluate glomerular and tubular renal functions and analyze blood pressure in a cohort of pediatric patients with juvenile idiopathic arthritis (JIA).

**Methods:**

A total of 40 pediatric patients, 20 (50%) with JIA and 20 (50%) healthy control subjects, were studied, and performed the renal function on 24-h collection and the 24-h ambulatory blood pressure monitoring (ABPM). Moreover, we compared renal function and blood pressure trends between the groups of JIA patients with different disease activities.

**Results:**

No statistically significant differences were observed between patients with JIA and healthy children in terms of glomerular filtration rate (GFR), fractional excretion of sodium (FENa), tubular reabsorption of phosphate (TRP), and calcium-creatinine urine ratio (CaU/CrU). In contrast, we observed significantly higher values in JIA patients than in controls for the presence of hematuria (*p* < 0.0001) and proteinuria (*p* < 0.0001). Compared to the control group there were significantly higher values of hematuria and proteinuria/day in both groups of JIA patients with low disease activity (respectively, *p* = 0.0001 and *p* = 0.0002) and moderate disease activity (respectively *p* = 0.0001 and *p* = 0.0012). Systolic and diastolic dipping were significantly reduced in patients with JIA compared with healthy controls (*p* < 0.0001 and *p* < 0.0001, respectively).

**Conclusions:**

Our study showed that children with JIA, already in the early stages of the disease, have higher values of hematuria and proteinuria, which are early warning signs of nephropathy. Therefore, detailed screening of renal function and pressure monitoring in patients are necessary to monitor their evolution over time.

## 1 Introduction

Juvenile idiopathic arthritis (JIA) is the most common rheumatic disease in childhood (1, 2) and according to the International League of Associations for Rheumatology (ILAR) classification seven JIA categories can be identified, with distinct clinical symptoms and disease outcomes (3).

In addition to pathognomonic joint disease, JIA is also burdened by extra-articular involvement and comorbidities (4–6), the most frequent of which are uveitis and cardiovascular complications (7–9). However, the pathogenesis of renal involvement in JIA is poorly understood, it may develop because of subclinical endothelial damage due to the inflammatory systemic process itself, leading to kidney disease, cardiovascular disease up to hypertension in children with JIA.

Despite the known correlation between chronic inflammation and risk of early endothelial dysfunction and renal damage described in adults with RA (rheumatoid arthritis) (10), there are only few published data assessing the renal involvement risk in children with JIA (11, 12).

In fact, there is no evidence in literature about an extensive evaluation of renal function and 24-h ambulatory blood pressure monitoring (ABPM) in children affected by JIA.

Therefore, the aim of this study was to evaluate whether JIA children show signs of early renal damage as glomerular and tubular renal function and analyze blood pressure compared to healthy controls and to establish the possible relationship with disease activity in JIA patients.

## 2 Materials and methods

### 2.1 Study design and setting

This non-randomized controlled case-control study followed the STROBE guidelines (13). This study was conducted at the Pediatric Unit of Santa Maria Goretti Hospital, Latina Polo Pontino Sapienza University of Rome.

Informed written consent was obtained from the parents or caregivers of each child included in the study. The study was approved by the local ethics committee, and the protocol conformed to the ethical guidelines of the 1975 Declaration of Helsinki, as revised in 2000 (14).

### 2.2 Participants

The study cohort included children with JIA (group JIA) at the time of fist JIA diagnosis who were not yet on therapy and a control group of healthy children (HC group) referred to our pediatric rheumatology outpatient clinic.

JIA was diagnosed based on the International League of Associations for Rheumatology (ILAR) criteria (2). The HC group included patients evaluated for joint pain without any comorbidities, with the exclusion criteria of a JIA diagnosis or other inflammatory rheumatologic disorders, and not taking any type of medication.

Exclusion criteria for all cohort included children with any malignancy and comorbidities; history of chronic metabolic, infectious, and other systemic diseases; food allergies and malabsorption; obesity [body mass index (BMI) > 85° percentile]; previous treatment with drugs potentially affecting renal function; documented orthostatic proteinuria; congenital anomalies of the urinary tract; and known nephrological disorders.

### 2.3 Variables, interventions and measures

During clinical evaluation, demographic data, weight, height, and BMI were collected for each subject.

#### 2.3.1 Laboratory measures

At the time of physical examination, all children underwent 24-h urine collection done at home, according to our pediatric nephrology section, following the instructions given to the parents and children by trained nurses or doctors to ensure proper collection as for standard protocol routinely used. All subjects underwent laboratory investigations, including albumin, platelets, hemoglobin, antinuclear antibodies (ANA), inflammatory indices such as C-reactive protein (CPR), erythrocyte sedimentation rate (ESR), serum creatinine, sodium, and phosphorus in the blood sample. Moreover, creatinine, sodium, phosphorus, calcium, and proteinuria were analyzed in 24-h urine collections (15).

For the study of renal function, the glomerular filtration rate (GFR) was calculated as creatinine clearance [using the formula (U)_cr _× Urine volume_ml_/Time_minutes_/(S)_cr_, normalized to body surface area (assumed as normal if GFR > 90 ml/min/1.73 m^2^)], proteinuria (mg/m^2^/24 h), (normal value if <100 mg/m^2^/24 h) (16–18) and urinary creatinine excretion (mg/kg body weight). The fractional excretion of sodium (FENa), tubular reabsorption of phosphate (TRP), and calcium-creatinine urine ratio (CaU/CrU) were also evaluated. We considered normal values: FENa < 1%, TRP > 85%, CaU/CrU < 0.8 mg/mg in infants <6 months, <0.6 mg/mg between 6 and 12 months and <0.2 in older children. The presence of microhematuria was investigated using urine cytofluorometry (n.v. < 10 RBC/µl).

#### 2.3.2 Blood pressure measurements

In controls and patients, we also studied 24 h blood pressure (BP) trends using a blood pressure monitoring (ABPM), for this purpose, the parents and children were instructed by an experienced nurse in keeping a diary of the child's physical activity during the 24 h of the monitoring (19). Therefore, we applied to all children a 24-h ABPM using a Spacelabs Healthcare model apparatus (OnTrak) with a properly sized cuff on the nondominant arm following the technical modalities described in the approved standard guidelines (19). Readings were performed every 20 min during the day (7 A.M.–10 P.M.) and every 30 min at night (10 P.M.–7 A.M.). Reference values for ABPM readings are those published by the American Heart Association (19) staging in (1) normal BP, (2) white-coat hypertension, (3) masked hypertension, and (4) ambulatory hypertension. Moreover, we calculated the dipping for both systolic and diastolic arterial blood pressure, regarded as normal, by 10%–20% (19).

#### 2.3.3 Demographic and disease activity patients' findings

For patients with JIA, the following variables were recorded and assessed for each patient: disease related variables including JIA disease subtype, age at disease onset, the number of active and limited joints assessed by the physician, the physician's global assessment of disease activity (PhGA) expressed on a 21-circled scale (0–10 in increments of 0.5, where 0 is no activity and 10 maximum activity), and the Juvenile Arthritis Disease Activity Score (JADAS)-10, and clinical JADAS (cJADAS)-10. The JADAS is a continuous disease activity score specific to JIA and consists of 4 variables: the PhGA, patient (or parent) global assessment (PtGA) of overall well-being (expressed on a 21-circled scale of 0–10 in increments of 0.5), counts of active joints (assessed up a maximum value of 10 for the JADAS-10) and the erythrocyte sedimentation rate (ESR). The cJADAS-10 is the JADAS-10 excluding the evaluation of the ESR (20–22).

Children with JIA were classified in agreement with the cJADAS 10, considering as the most feasible scoring system, in groups with low, moderate, or high disease activity (23, 24).

### 2.4 Outcomes

The primary objective of this study was to test whether renal function and blood pressure trends were normal in children with JIA compared with those in the control group.

The secondary objective was to compare renal function and blood pressure trends between groups of subjects with different disease activities.

### 2.5 Statistical analysis

Statistical evaluation was performed using the dedicated software: JMP 15.2.1 for MacOs (SAS Institute Inc. Nominal variables were compared using the chi-squared (*χ*^2^) test or Fisher's test. For all the parameters considered in the study the approximation to normal of the distribution of the population was tested by Kolmogorov–Smirnov One-Sample Test and statistics for kurtosis and symmetry. As results were asymmetrically distributed, data are expressed as median and interquartile range (IQR), 25th and 75th quartile, and non-parametric tests were used. Data were analyzed with the Kruskal–Wallis nonparametric one-way analysis of variance to examine the changes of parameters in both groups related to JIA disease activity. The null hypothesis was that the groups of the study all came from the same distribution. When the Kruskal–Wallis test was significant, we used the Wilcoxon test to compare the intragroup differences at the five observation times. Moreover, in case of statistical significance, the statistical power was calculated. Parametric data were expressed as mean ± standard deviation (SD) and analyzed with student test. Categorical data were expressed as frequencies and percentages. Statistical significance was set at *p*-value <0.05.

## 3 Results

### 3.1 Demographic and study group

We identified a total of 52 children, 32 (61.54%) with JIA and 20 (38.46%) healthy control children, 12 (23%) JIA patients were excluded because they did not perform renal function tests on 24-h collection or 24-h ABPM. Therefore, a total of 40 pediatric patients who met our inclusion criteria were included in the analysis: 20 (50%) with JIA and 20 (50%) healthy control subjects.

The baseline characteristics of the cohort are presented in Table 1. No statistically significant differences were found in the demographic features between patients with JIA and controls.

**Table 1:** Baseline characteristics of participants.

	JIA (*n* = 20)	HC (*n* = 20)	*p*
Age, years	16 (11.5–17.8)	15 (9.8; 17)	NS
Male, *n* (%)	5 (12.5%)	5 (12.5%)	NS
Weight, [kg]	53.4 (37–59)	53.2 (37.3–59.4)	NS
Weight, [percentile]	38.9 (10.6–79.1)	67.1 (52.3–74.7)	NS
Height, [cm]	159.5 (145.5–164.8)	161 (141.3–167.1)	NS
Height, [percentile]	53.6 (28.0–85.7)	55.7 (28.5–74.9)	NS
BMI, [kg/m^2^]	21.5 (17.1–23.5)	20.9 (18.7–22.1)	NS
Oligoarthritis, *n* (%)	15 (75%)	–	
Polyarthritis, *n* (%)	3 (15%)	–	
Psoriatic arthritis, *n* (%)	1 (5%)	–	
Enthesitis-related arthritis, *n* (%)	1 (5%)	–	
Systemic arthritis, *n* (%)	–	–	
Physician global assessment score, mean ± SD	1.75 ± 1.83	–	** **
Patient global assessment score, mean ± SD	1.95 ± 2.1	–	** **
c-JADAS 10, mean ± SD	4.1 ± 4.2	–	** **
Low disease activity, *n* (%)	12 (60%)	–	** **
Moderate disease activity, *n* (%)	8 (40%)	–	** **
High disease activity, *n* (%)	0	–	** **
ANA positive, *n* (%)	7 (35%)	–	
ESR, [mm/h]	6.5 (3.25–13.50)	1 (0–2.75)	NS
CRP, [mg/dl]	0.1 (0.03–0.65)	0 (0–0.05)	NS
Albumin, [g/L]) mean ± SD	4.5 ± 0.3	4.3 ± 0.5	NS
Platelets, [cell ×1.000/mm^3^] mean ± SD	264.1 ± 72.8	273.2 ± 54.21	NS
Hemoglobin, [g/dl] mean ± SD	13.35 ± 1.48	12.55 ± 0.8	NS

Except where indicated otherwise, values are expressed as median (1st–3rd quartile).

The JIA cohort was characterized by a high frequency of oligoarthritis 15 (75%) according to ILAR criteria. As shown in Table 1, 12 (60%) and 8 (40%) of JIA patients presented respectively with low and moderate disease activity, according to the ACR recommendations.

### 3.2 Laboratory findings

No statistically significant differences were observed between JIA patients and healthy children in Glomerular Filtration Rate (GFR) [121.3 (90.4–146.5) ml/min/1.73 m^2^ vs. 125 (116–146.8) ml/min/1.73 m^2^, *p* NS]; FENa [0.7 (0.5–0.7) % vs. 0.7 (0.6–0.8) %, *p* 0.8], TRP [90.4 (88.2–92.7)% vs. 90.4 (89–92.5)%, *p* NS], CaU/CrU [0.1 (0.1–0.2) mg/mg vs. 0.1 (0.1–0.2) mg/mg, *p* NS] (Table 2).

**Table 2:** Glomerular and tubular renal function on 24-h collection in JIA patients and controls.

	JIA (*n* = 20)	HC (*n* = 20)	*p*	Power analysis
FENa, %	0.7 (0.5–0.7)	0.7 (0.6–0.8)	NS	–
TRP, %	90.44 (88.2–92.7)	90.44 (89–92.5)	NS	–
CaU/CrU, [mg/mg]	0.1 (0.1–0.2)	0.1 (0.1–0.2)	NS	–
GFR_Cr,_ [ml/min/1.73 m^2^]	121.3 (90.4–146.5)	125 (116–146.8)	NS	–
Hematuria, [RBC/µl]	3.5 (2–8.5)	0.5 (0–2)	<0.0001	0.95
Proteinuria, [mg/m^2^/die]	92.5 (78.1–118.3)	49.5 (24–68)	<0.0001	0.57

FENa, fractional excretion of sodium; TRP, tubular reabsorption of phosphate; CaU/CrU, calcium-creatinine urine ratio; GFR, glomerular filtration rate; NS, not significant.

Values are expressed as median (1st–3rd quartile).

In contrast, we showed significantly higher values in JIA patients than in controls for the presence of hematuria [3.5 (8.5–2) RBC/ul vs. 0.5 (0–2) RBC/ul (*p* < 0.0001)] and proteinuria [92.5 (78.1–118.3) mg/m^2^/24 h vs. 49.5 (23.4–68.0) mg/m^2^/24 h; *p* < 0.0001].

### 3.3 Glomerular and tubular renal function related to disease activity in JIA

**Figure 1:**
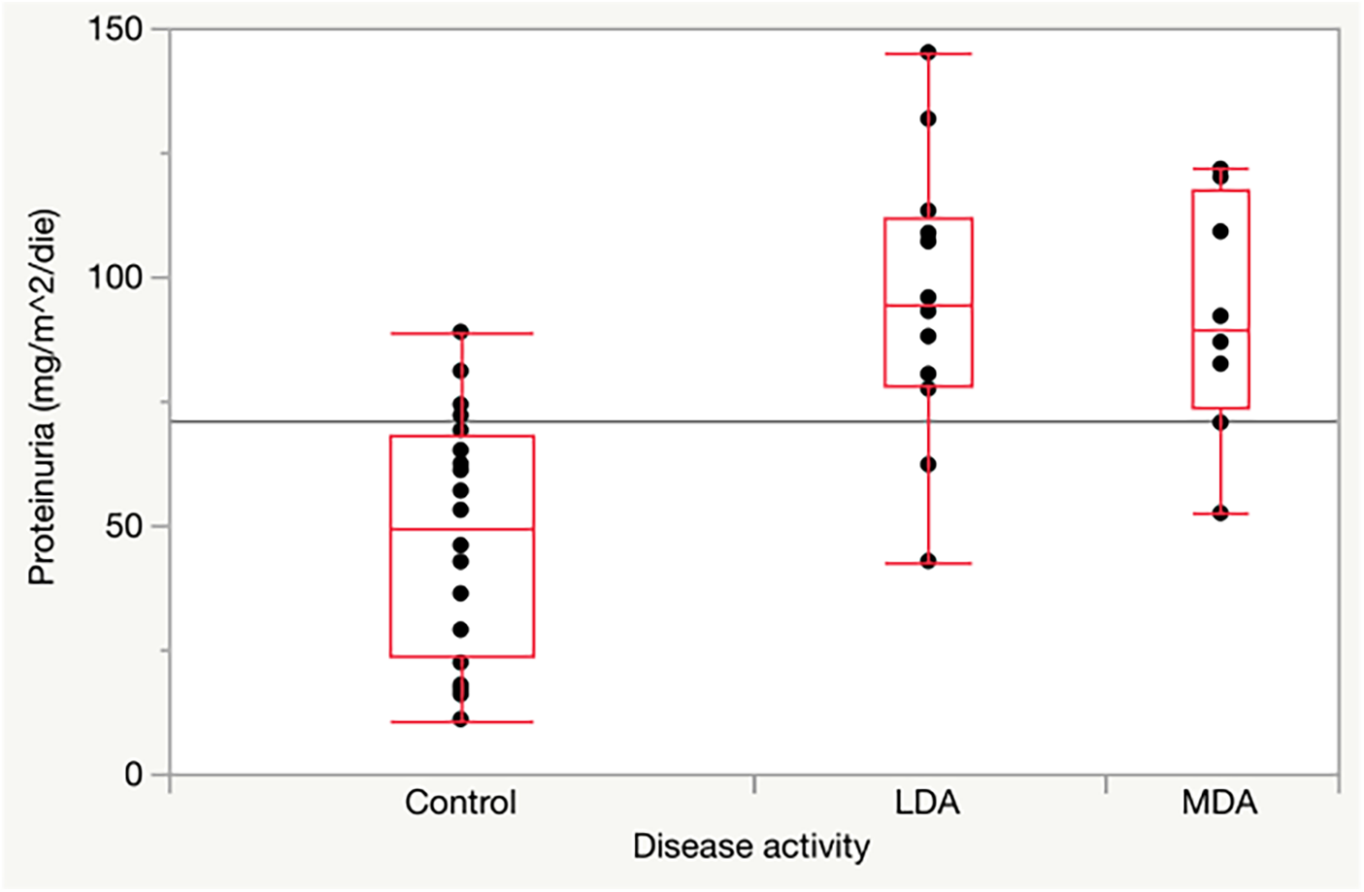
Proteinuria in JIA children with low and moderate disease activity compared to healthy controls.

As shown in Table 3, there were no significant differences in glomerular and tubular functions between the different disease activity JIA subgroups. Conversely, compared with the control group, there were significantly higher values of hematuria and proteinuria/day in both groups of JIA patients with low disease activity (*p* =** **0.0001 and *p* = 0.0002, respectively) and moderate disease activity (*p* =** **0.0001 and *p* = 0.0012, respectively) (Figure 1).

**Table 3:** Glomerular and tubular renal function on 24-h collection in JIA groups based on disease activity and controls.

	JIA LDA	JIA MDA	HC	Kruskal–Wallis *test p*	Power analysis
FENa, [%]	0.6 (0.5–0.7)	0.7 (0.5–0.7)	0.7 (0.6–0.8)	NS	–
TRP, [%]	90.4 (89.1–91.0)	90.9 (87.5–94.2)	90.44 (89–92.5)	NS	–
CaU/CrU, [mg/mg]	0.1 (0.1–0.2)	0.1 (0.1–0.1)	0.1 (0.1–0.2)	NS	–
GFR_Cr,_ [ml/min/1.73 m^2^]	126.5 (107.5–143.7)	95.7 (88.5–148)	125 (116–136.6)	NS	–
Hematuria, [RBC/ul]	3.5 (2.25–8)	10.8 (16–1.25)	0.5 (2–0)	0.0004	0.89
Proteinuria, [mg/m^2^/die]	94.36 (78.12–127)	89.4 (117.3–73.5)	49.5 (23.9–68)	<0.0001	0.61
Wilcoxon test, *p*	JIA LDA vs. HC	Power analysis	JIA MDA vs. HC	Power analysis	JIA LDA vs. MDA.
Hematuria	0.0001	0.91	0.0001	0.95	NS
Proteinuria	0.0002	0.48	0.0012	0.68	NS

FENa, fractional excretion of sodium; TRP, tubular reabsorption of phosphate; CaU/CrU, calcium-creatinine urine ratio; GFR, glomerular filtration rate; LDA, low disease activity; MDA, moderate disease activity; NS, not significant.

Values are expressed as median (1st–3rd quartile).

### 3.4 Blood pressure related to disease activity in JIA

No differences were found in ambulatory blood pressure monitoring classification (19) between children with JIA and controls. Instead, systolic and diastolic dipping were significantly reduced in patients with JIA compared with healthy control subjects (systolic 7.8 ± 5.4% vs. 21.37 ± 7.6% *p* < 0.0001; diastolic 12.4 ± 9.7% vs. 20.65 ± 5.6% *p* < 0.0001).

## 4 Discussion

Rheumatological diseases, being systemic diseases, may also have renal involvement, as described mainly in connective tissue disease and vasculitis (10). The pathogenetic mechanisms of these nephropathies are still unclear and may be due to circulating autoantibodies, as described in rheumatoid arthritis (RA), which target podocyte proteins causing membranous nephropathy as well as an endothelial dysfunction linked to inflammation that have been described in children and adults with JIA (8, 25). Specifically, renal disorders could be sequelae of chronic systemic inflammation, causing involvement of the endothelium of the glomerular capillaries, which may result in the onset of renal glomerular disease.

In addition, there are possible undesirable effects of antirheumatic treatments. In fact, some therapy-related cases have been described where kidney damage disappeared after discontinuation of the drug involved (10). Otherwise, it should also be considered that opinions on this matter may be conflicting. In fact, in adult patients with RA, renal damage, initially described as a consequence of drug therapy, is now perhaps considered by some authors to be related to the disease itself (26–30). For this reason or also due of a possible interference caused by drugs, we considered children at first diagnosis who had not yet started any therapy, according to a common cause of kidney injury. Our study showed that children with JIA, already in the early stages of the disease and before the start of therapy, had higher values of hematuria and proteinuria, which are early warning signs of nephropathy. Data that might be in agreement with the study of Cafarotti et al. (11) described a reduced eGFR in JIA patients attributable to disease progression. We believe that these early signs of renal damage could be attributed to proinflammatory stimuli that could directly contribute to vascular inflammation (31) causing renal vasculitis (32), similar to what has been described in cases of vasculitis mainly associated with systemic juvenile idiopathic arthritis (33). All our patients are currently on nephrological follow-up to monitor the possible persistence of renal alterations and check if, in the future, a renal biopsy will be necessary to detect any progressive renal involvement.

Moreover, our study did not describe differences in systolic and diastolic arterial blood pressure with respect to healthy children at ambulatory blood pressure control as well as at the ABPM 24 h check, but only a reduction in both systolic and diastolic dipping, as it may be associated with an increased risk of adverse cardiovascular outcomes in adulthood (19). Conversely, Gicchino et al. showed that 8.2% of JIA children could present hypertension at ambulatory measurement (34). The role of dipping as an early sign of cardiovascular disease has yet to be well defined and we would like to continue a follow up of these patients to accurately characterize the evolution.

Our study had some limitations, first has a limited number of patients as well as a relatively small number of children for each JIA subtype. Moreover, further longitudinal studies with larger patient groups are needed also to study these patients in follow up. Despite these limitations, our data highlight the importance of performing detailed screening of renal function and pressure monitoring in patients with JIA. Regular screening for proteinuria and hematuria in children with JIA might detect early renal damage, which is the most common initial presentation of kidney disease. In fact, these children could benefit from early treatment with ace inhibitors possibly associated with sartans which, by promoting a reduction in proteinuria, slow down the non-immunological progression of renal damage as described by Lubrano et al. (35). It should also be considered that the early use of this therapy has positive effects on the heart (35), which is one of the main organ targets of this pathology.

## 5 Conclusions

In conclusion, children with JIA could have a kidney function and blood pressure alterations compared to healthy controls indicating a potential complication of disease. Therefore, a periodic screening is suggested in JIA patients.

We also believe that larger longitudinal studies in these children are necessary to confirm preliminary data and their evolution over time, while evaluating the possibility of starting a renal function protection therapy at an early stage of disease.

## Data Availability

All relevant data are reported in the article. Additional details can be provided by the corresponding author upon reasonable request.
